# Immunosuppressant-Responsive Enteropathy and Non-Responsive Enteropathy in Dogs: Prognostic Factors, Short- and Long-Term Follow Up

**DOI:** 10.3390/ani11092637

**Published:** 2021-09-08

**Authors:** Elena Benvenuti, Alessio Pierini, Enrico Bottero, Marco Pietra, Eleonora Gori, Stefano Salvadori, Veronica Marchetti

**Affiliations:** 1 Department of Veterinary Science, University of Pisa, Via Livornese Lato Monte, 56122 Pisa, Italy; elebenve81@gmail.com (E.B.); pierini.alessio2004@libero.it (A.P.); veronica.marchetti@unipi.it (V.M.); 2 Endovet Italia, Via Antonio Oroboni, 00100 Rome, Italy; botvet@alice.it; 3 Department of Veterinary Clinical Sciences, University of Bologna, Via Tolara di Sopra, 50, 40064 Bologna, Italy; marco.pietra@unibo.it; 4 Institute of Clinical Physiology, National Research Council (CNR), Via Moruzzi 1, 56124 Pisa, Italy; stefsa@ifc.cnr.it

**Keywords:** chronic enteropathy, prognosis, CCECAI, response, relapse, outcome, canine

## Abstract

**Simple Summary:**

Chronic intestinal inflammation in dogs is a challenging disease to manage. Most studies about prognostic factors and follow-up data are only available for small populations or with short-term follow-up. The aim of this study of 165 dogs with chronic intestinal inflammation was to identify clinical and haematological factors associated with mortality, clinical response and relapse, with long-term follow-up. Nine per cent of dogs did not respond to therapy at 1 month follow-up. Most dogs with chronic intestinal inflammation had a good clinical course in most cases, and the non-response or relapse rate was 9–11%. A reduction of body condition (loss of weight), lower serum albumin concentration and presence of lacteal dilatation on intestinal histology at diagnosis were identified as factors associated with a decreased response rate, higher mortality and lower chance of achieving long-term remission.

**Abstract:**

A multicentre prospective study was performed to assess whether clinical, hematobiochemical, endoscopic and histopathological parameters were associated with mortality, clinical response and relapse of disease in short- and long-term follow-up of a total of 165 dogs with chronic inflammatory enteropathy, of which 150 had immunosuppressant responsive enteropathy (IRE), and 15 had non-responsive enteropathy (NRE) dogs. Clinical severity (CCECAI) was evaluated from presentation (T0) to 18 months (T18) from diagnosis. T0 body condition score (BCS), selected haematological parameters and endoscopic and histopathological scores were evaluated. Presence/absence of histopathological duodenal lesions was recorded. Responders were evaluated using CCECAI at T1. Relapse was evaluated from T3 to T18. Long-term responders included dogs who responded at T1 and showed no relapse. Dogs were divided into responders/non-responders, survivors/non-survivors and relapsed/non-relapsed. At T1, 15/165 dogs (9%) were considered NRE. Sixteen dogs (11%) were considered relapsed at T3, 8% at T6 and 10% at T12, and none of 96 dogs relapsed at T18. NREs showed significantly lower BCS than IREs. Non-survivors showed a significantly lower serum albumin concentration and BCS than survivors. Non-responders, relapsed or non-survivors had higher presence of lacteal dilatation compared to long-term responders. Dogs with IRE showed a good clinical course with a low relapse rate, with only a few dogs in the NRE group. Reduction of BCS, albumin and lacteal dilatation at diagnosis may be considered negative prognostic factors for response, mortality and long-term disease remission.

## 1. Introduction

Chronic enteropathy (CE) in dogs is defined as a complex interaction among host genetics, the intestinal microenvironment and the immune system [1]. Thus, a sequential therapy using specially formulated diets, probiotics, antimicrobials and immunosuppressive drugs is the most common strategy used to achieve clinical remission. Recently, Cerquetella et al. [2] proposed novel inclusion criteria in the diagnosis of IRE without the use of antibiotic trials after diet trials [2]. The final diagnosis is based on the response to treatment, histopathological evaluation of intestinal biopsy or all the above. Immunosuppressant-responsive enteropathy (IRE) is defined as an idiopathic, multifactorial intestinal inflammation in which diet and antibiotic trials have failed [3]. In addition, in IRE, intestinal inflammation must be demonstrated on histopathology, and response to an immunosuppressant therapy is needed [3,4]. Dogs not responding to immunosuppressant treatment are categorized as having non-responsive enteropathy (NRE) [3,4]. Protein-losing enteropathy (PLE) is a particular subtype of enteropathy with worse prognosis, which is classified based on the presence of low levels of albumin and the absence of other protein-losing disease (i.e., protein-losing nephropathy, protein-losing dermatopathy). The CE can be associated with PLE, and the potential causes are numerous. IRE, NRE and intestinal lymphangiectasia are considered the most common causes of PLE in dogs [5,6].

Several studies have evaluated potential prognostic factors in dogs diagnosed with CE or PLE. A serum albumin < 2 g/dL was associated with a negative outcome (euthanasia or non-response to treatment) within three years after diagnosis in a retrospective study on seventy dogs with CE [7]. In Volkmann et al. [8], in 97 dogs with CE, serum albumin and cobalamin concentrations were significantly lower in dogs with no remission of symptoms than in dogs that responded to treatment [8]. Serum albumin, total protein and cholesterol values were significantly lower in dogs with PLE that died within six months from diagnosis than those dogs that survived longer [9]. Other authors [10,11,12], studied clinical scores in dogs with PLE. In this class of patients, a higher CIBDAI or CCECAI at diagnosis, or their lack of decrease during treatment, was associated with higher risk of death [9,10,11].

As a chronic waxing and waning disease, IRE can be very difficult to manage, and only a few studies have investigated potential prognostic factors associated with the response in dogs with IRE [1,4,13,14]. Moreover, in Heilmann’s study, among the 127 included dogs with CE [14], only 17 dogs were finally diagnosed with IRE, and only two dogs diagnosed with IRE were included in long-term follow-up in 19 dogs with CE in Dandrieux’s study [4]. In Heilmann et al.’s study [14], the authors classified the clinical response as complete, partial or absent (non-response), based on the difference between Canine Chronic Enteropathy Clinical Activity Index (ΔCCECAI) before and after therapy. Seventeen dogs with SRE/IRE had significantly higher CCECAI scores than dogs with FRE/ARE. In the SRE/IRE group, 12 dogs were completely responsive, 3 partially responsive and 2 nonresponsive [14]. To date, there are only a few studies on the prospective evaluation of the short- and long-term follow-up of IRE and NRE dogs [4,13,14].

The aim of the present study was therefore to prospectively evaluate dogs with IRE, verifying whether clinical, hematobiochemical, endoscopic and histopathological parameters evaluated at presentation were associated with response to treatment, mortality or relapse of the disease, in short- and long-term follow-up.

## 2. Materials and Methods

This prospective multicentre study was performed at two university veterinary hospitals (Pisa and Bologna universities) and at an Italian private veterinary clinic between January 2017 and September 2019. Blood samples required for haematological tests were taken after obtaining the owners’ informed consent, and the study received the official approval of the animal welfare committee of the University of Pisa (OPBA number 31834/2017).

Dogs with presumptive IRE diagnosis were included in this study. The dogs included in this study showed chronic gastrointestinal disease (including weight loss, vomiting, diarrhoea, decreased appetite) for at least three weeks. Clinical evaluation was performed, including haematobiochemical panel, urinalysis including urinary protein-to-creatinine ratio, faecal flotation, Giardia antigen test, trypsin-like immunoreactivity (TLI) and serum basal cortisol to exclude enteric parasites, pancreatic failure and endocrine or kidney diseases. All dogs had a full abdominal ultrasound examination performed at the time of the inclusion in the study to exclude extra-intestinal disease or non-primitive inflammatory intestinal disease (e.g., intussusception, foreign bodies, or intestinal tumours). Furthermore, dogs were also eligible to be included in the IRE group if they failed a diet trial with a monoprotein and/or hydrolysed (HA Purina^®^, Nestlé Purina S.p.a. Assago, (MI) Italy or Z/d Hill’s^®^, Hill’s Pet Nutrition Italia S.R.L. Rome, Italy) diet of at least 2 weeks.

ARE was also excluded, at the discretion of clinicians, through antibiotic trial with tylosin at 15 mg/kg every 12 h for 3 weeks [1,3,15]. Dogs with FRE and ARE were therefore excluded from the study before endoscopy and histopathology exams of gastro enteric samples [3,16].

For each dog included in the study, an immunomodulatory therapy was started using prednisolone (164 dogs, dosage ranged from 0.5 to 1 mg/kg every 12 h, Prednicortone^®^, Dechra Veterinary Products S.r.l., Turin, Italy) or budesonide (1 dog, 3 mg/m^2^ every 24 h, Intesticort^®^, Sofar S.p.A, Bologna, Italy) in association with cyclosporine (38 dogs, 5 mg/kg every 24 h, Atoplus^®^, Elanco Italia S.p.A, Sesto Fiorentino, Florence, Italy) or chlorambucil (23 dogs, 2–4 mg/m^2^, Leukeran^®^, Aspen Pharma Trading Limited, Lake Drive, Citywest, Dublin, Ireland) at the clinicians’ discretion. In all dogs, during the diet trial and the immunosuppressant therapy, a multistrain probiotic was used. In dogs with hypocobalaminaemia (evaluated with a competitive chemiluminescent enzyme immunoassay (Immulite 1000, Diagnostic Products Corporation, Los Angeles, CA, USA), cobalamin was supplemented SC every week (250–500 mcgr/dog, Dobetin B1^®^, Ecuphar Italia S.r.l. Milano, Italy).

The time points of the present study are reported with the letter T followed by a number referring to the time of clinical rechecks expressed in months (i.e., T1 = clinical recheck at 1 month). At T0, the dogs started immunomodulatory therapy, and T0 coincides with the day after histopathological exam.

In all dogs, clinical evaluation was performed, and clinical severity was evaluated using the previously published and validated CCECAI score at T0, T1, T3, T6, T12 and T18 [7]. The CCECAI scoring system assessed 9 categories of clinical signs, including attitude and activity, appetite, vomiting, faecal consistency, faecal frequency, weight loss, serum albumin concentration, peripheral oedema and ascites, and pruritus [7] (Table S1). The initial clinical evaluation and all the clinical rechecks of each dog were performed by the same clinician that included the dog in the study.

The body condition score (BCS, on a 1–9-point scale) [17] was also evaluated at T0. In addition, total proteins (TP, laboratory reference interval 5.8–7.8 g/dL), albumin (ALB, laboratory reference interval 2.7–4.1 g/dL), cholesterol (COL, laboratory reference interval 120–280 mg/dL) and C-reactive protein (CRP, laboratory reference interval 0–0.3 mg/dL) were analysed at the clinical pathology laboratory of the University of Pisa with an automated biochemistry analyser (Lyasis, Assel srl, Rome). Serum samples were stored at −80 °C for a maximum of 6 months. The presence of protein losing enteropathy (PLE) was defined if serum ALB concentration was lower than 2.7 g/dL [10].

Gastrointestinal endoscopy was performed by well-trained endoscopists (more than 15 years’ experience) using Fujinon EG-200FP and EG-270NS (Fujinon Corporation, Saitama, Japan). Each dog underwent general anaesthesia for endoscopic examination of the gastrointestinal tract and endoscopic biopsy of the stomach, duodenum and colon, with at least 8 biopsy samples obtained from each region. In addition, in all cases in which the access to ileum was possible, this region was also explored, and biopsy specimens were acquired. Each gastrointestinal tract was graded for alterations following the World Small Animal Veterinary Association Gastrointestinal Standardization Group (WSAVA) guidelines [18]. Endoscopic scores ranged from normal (0), mild (1), moderate (2), to severe (3). Intestinal biopsies were performed by endoscopy using 2.4 mm oval clamshell biopsy forceps (Fujinon EG-200FP and EG-270NS, Fujinon Corporation, Saitama, Japan), and histopathological biopsies of each gastrointestinal tract were evaluated according to the guidelines of the WSAVA, and severity of inflammation was scored from normal (0), mild (1), moderate (2), to severe (3) [19]. Histopathological specimens were evaluated by independent pathologists (European College Veterinary Pathology diplomate or 20-years experienced pathologists). Although all the digestive tract specimens were evaluated to reach a final diagnosis for each dog, only endoscopic and histopathological scores of duodena were used for the statistical analysis.

The presence (score 1) or absence (score 0) of histopathological lesions, such as crypt dilatation (CD), intraepithelial lymphocytes (IEL), mucosal fibrosis (MF) and lacteal dilatation (LD), was histologically assessed on duodenum samples. The sum (SUM) of the histopathological lesions reported above was calculated.

Responder dogs were evaluated comparing CCECAI score at T1 with T0; a reduction in CCECAI ≥ 75% was classified as a complete response, a reduction in CCECAI between >25% and <75% as a partial response, and as a non-response if dogs had a reduction in CCECAI ≤ 25%. Relapse was evaluated from T3 to T18. Delta CCECAI (ΔCCECAI) was calculated for each dog at any time point as the difference between the CCECAI of interest and the immediate previous CCECAI. Dogs with a ΔCCECAI ≥ 2 were considered relapsing and assigned to the relapse group. In addition, dogs that died between T1 and the end of the study were recorded as having relapsed at the next scheduled assessment point following their death (e.g., a dog that died 4 months after T0 was considered to have relapsed at T6) [20]. Dogs with CCECAI of interest < 3 were considered not relapsed even if CCECAI was higher than the previous CCECAI.

The non-responders/relapsed group also included non-survivors, which died or were euthanized due to disease progression. During the study, no dogs died from causes unrelated to IRE. The long-term follow-up was evaluated as follows: responder dogs at T1 and dogs did not relapse up to the last follow-up (T18) were included in the “long-term responders” group. All the other dogs (dogs showing no response at T1 or relapsing during the 18-month follow-up or died within the study period) were assigned to the group “non-responders, relapsed, non-survivors”.

### Statistical Analysis

Data were presented as follows: mean ± SD for normally distributed data; median and range (min–max) for non-normally distributed data; absolute and relative frequency for categorical data. Predictors included BCS, TP, ALB, COL, CRP, endoscopic and histopathological scores, CCECAI T0, CD, IEL, MF, LD and SUM. Response at T1 (responders/non-responders), relapse, mortality (alive/non-survivors) and long-term response (long-term responders/non-responders-relapsed-non-survivors) were considered outcomes (Table 1). The Lilliefors (Kolmogorov–Smirnov) test for normality was initially used to evaluate the data distribution of all continuous parameters (age, TP, ALB, COL and CRP). Sex and sexual status, endoscopic and histopathological scores, the presence/absence of CD, LD, MF and IEL were analysed as categorical data. The BCS, TP, ALB, COL, CRP, SUM and CCECAIT0 were assessed for a correlation with each other with Spearman’s correlation tests. Before developing multivariable models, each potential predictor was evaluated for its association with each outcome using univariate logistic regression models. Multivariate logistic regression models were used to identify potential predictors that were significant (*p* < 0.05) in univariate analysis. Pairwise interactions and multicollinearity between final model variables were assessed. Predictors were removed one to one from the final model either if pairwise interactions were significant (*p* < 0.05) or if variance inflation factors (VIF) were higher than 5. Non-independent variables (TP, CCECAIT0, SUM) were not included in the multivariate logistic regression models.

A receiver operating characteristic (ROC) analysis was performed and the area under the curve (AUC) and the 95% confidence intervals (95% CI) were calculated. ROC curves were used to optimize decision thresholds to distinguish predictors significantly associated with each outcome at the final model. Diagnostic thresholds and their sensitivity and specificity were determined according to the maximum Youden index.

Dogs lost during the follow-up together with dogs with missing information were excluded from the statistical analysis. Commercial software was used to analyse the data (SPSS v. 23, IBM Corp., Armonk, NY, USA). A *p*-value of <0.05 was considered significant in all the tests.

## 3. Results

The present study included 165 dogs. Descriptive statistics are summarized in Table 2.

All the histopathology samples were adequate for all the subjects included in the study. In 12 patients (7%), no histopathological lesions were found, while in 54/165 dogs (33%) only one alteration was found. In 64/165 (39%) and 24/165 (15%) dogs, two and three histopathological lesions were simultaneously found, respectively. Eight of 165 dogs (5%) presented all four histopathological lesions. Lastly, in three (2%) dogs, only an inflammatory infiltrate without the presence of the aforementioned histopathological lesions was found.

**Figure 1:**
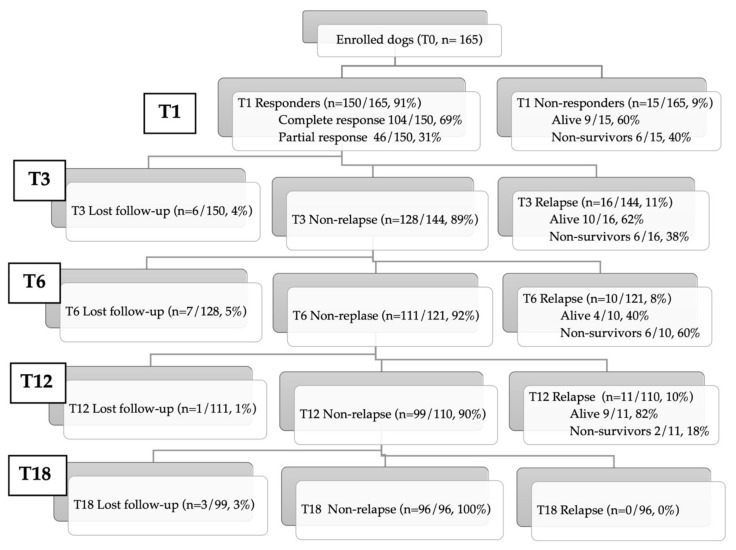
Flow diagram of inclusion (T0; 165 dogs) and clinical follow-up at various timepoints (T1, T3, T6, T12, T18 months) in immunosuppressant-responsive and non-responsive enteropathy dogs.

At T0, the median CCECAI was 8 (range 3–16), and at T1, the median CCECAI was 0 (range 0–14) in 159 dogs. Six of 165 dogs (4%) died within T1 and were not evaluable for CCECAI. Regarding the response at T1, an overall response rate of 91% (150/165) was observed. One-hundred-four of 165 dogs (63%) had a complete response, 46/165 dogs (28%) had a partial response, whereas 15/165 dogs (9%) were considered NRE (of which 6 died). During the 18-month study period, follow-up information was lost in 17 of 165 (10%) cases. Sixteen of 144 (11%) dogs were considered relapsed at T3 (of which six died), 10/121 (8%) at T6 (of which six died), 11/110 dogs (10%) at T12 (of which three died) and none of 96 dogs at T18 (Figure 1). There was no statistically significant difference in relapse rates between complete responders and partial responder dogs.

There was a weak correlation between BCS and COL (*p* = 0.005 ρ = 0.218), BCS and SUM (*p* = 0.0001; ρ = −0.240), BCS and CCECAI (*p* = 0.012; ρ = −0.199), TP and SUM (*p* = 0.0001; ρ = −0.258), ALB and SUM (*p* = 0.002; ρ = −0.240), COL and CRP (*p* = 0.014; ρ = 0.193) and COL and SUM (*p* = 0.047; ρ = −0.156). There was a moderate correlation between BCS and TP (*p* = 0.0001; ρ = 0.317), BCS and ALB (*p* = 0.0001; ρ = 0.362) and COL and ALB (*p* = 0.0001; ρ = 0.582). There was a strong correlation between TP and ALB (*p* = 0.0001; ρ = 0.867), and TP and COL (*p* = 0.0001; ρ = 0.649). Correlations between continuous variables are shown in Table 3.

Among all the potential predictor variables tested for association with the outcomes using multivariable analysis, the following were statistically significant. In the multivariable analysis, the non-responders showed significantly lower BCS than responders (*p* = 0.01). The best cut-off of BCS predicting non-response was <4, with a sensitivity of 66% and a specificity of 87% (*p* < 0.001; AUC 0.851, 95%CI 0.75–0.95). Compared to survivors, non-survivors showed significantly lower ALB (*p* = 0.001) and BCS (*p* = 0.002). The best cut-off of BCS predicting mortality was <4, with a sensitivity of 70% and a specificity of 86% (*p* < 0.001; AUC 0.829, 95%CI 0.73–0.93). The best cut-off of ALB predicting mortality was <2.1 g/dL, with a sensitivity of 87% and a specificity of 71% (*p* < 0.001; AUC 0.82, 95%CI 0.72–0.92). When BCS and ALB were merged in a single predictor, dogs with both BCS < 4 and ALB < 2.1 g/dL had a 16,600% (*p* < 0.001; OR 166, 95%CI 18–1501) increased chance of death compared with dogs with a BCS ≥ 4 and ALB ≥ 2.1 g/dL. Regarding the long-term follow-up (18 months), the non-responders, relapsed or non-survivor dogs had more frequently the presence of LD (*p* = 0.008, OR 2.82, 95%CI 1.31–6.08) compared to dogs that experienced a long-term response (long- term responder group). No predictors were found independently associated with relapse.

## 4. Discussion

This novel study on prognostic factors involved a large number of dogs with IRE and NRE, in which a long-term follow-up is available. In this prospective multicentre study, the follow-up of each dog was assessed evaluating their response, signs of relapse, and mortality in the 18 months following diagnosis. In addition, analysing clinical, haematobiochemical, endoscopic and histopathological parameters, the BCS, ALB and LD were highlighted as potential predictors of response, mortality and relapse.

Several studies have tried to identify possible prognostic factors that characterize the clinical course of dogs with IRE. However, only a few are aimed at identifying factors that predict the response to therapy, especially in long-term follow-up [4,7,13,21].

In our population, only a small percentage of dogs appeared to be non-responders to immunomodulatory therapy (NRE). At each follow-up, we also found a relapse in the first 12 months after diagnosis, after which patients never seemed to relapse up to 18 months after diagnosis. The literature on the clinical follow-up of IRE dogs is very limited [4,13,14,22]. Recently, in 17 IRE patients, the clinical response was evaluated using the CCECAI score, and the clinical response was divided into complete, partial and non-response [14]. In Heilmann et al.’s study [14], the median follow-up was eight weeks (interquartile range 4–13 weeks), and 70% of dogs were responsive, and 18% were partial responders. A total of 12% were NRE, which was similar to our non-responder rate. Dandrieux and Mansfield [4] conducted a prospective study of chronic enteropathies and investigated their long-term follow-up (33.5 months) by assessing the response to therapy at two to three months. Unfortunately, only two cases of IRE were included in Dandrieux’s study, and both showed a remission of clinical signs, no relapses and a good response to therapy throughout the entire follow-up [4]. In Otoni’s study, a statistically significant reduction in the value of CIBDAI after treatment was highlighted. A total of 94% of dogs (*n* = 15/16) showed a positive clinical response (12 dogs were in complete clinical remission and 3 dogs in partial remission) while one dog did not respond (CIBDAI score increased from 6 to 8 before and after the first treatment) [13]. It is therefore difficult to compare these data with our study, in which a larger number of cases and follow-up timepoints (T1, T3, T6, T12, T18) were evaluated. To the best of our knowledge, no other prospective works have investigated the clinical outcomes of IRE-affected dogs with long-term follow-up and in which non-response and relapse were characterized.

The clinical activity index has been showed to be an important prognostic tool by several studies. In Nakashima and colleagues, a normalization of the CIBDAI (canine inflammatory bowel disease activity index) [23] within 50 days after the diagnosis was associated with a longer survival time [10]. In Kathrani et al., high values of CCECAI and serum urea at the time of diagnosis were associated with a higher risk of death in dogs with PLE, and it was shown that for each point of increase in CCECAI, the risk of death increased by 22.9% [11]. In contrast with Kathrani’s results, in other retrospective studies on patients with PLE, the clinical response or survival time was not significantly influenced by the activity indices at diagnosis [7,9,12]. However, in Gianella et al., a CCECAI score > 5 one month after the diagnosis appeared to predict mortality within six months after diagnosis [9].

Based on our results, the CCECAI, in multivariate analysis, is not predictive for clinical outcome, and the differences observed between our study and the studies described above could be linked to the different numbers and types of cases (only IRE dogs) and to the inclusion criteria and study design (prospective study and statistical analysis). Moreover, in the previously cited works, all the patients included were affected by PLE, unlike ours with IRE close to evenly distributed between PLE and non-PLE.

In our work, dogs included in the non-responders or non-survivors showed a significant reduction of BCS compared to responders and survivors, respectively. Moreover, the non-survivors showed a lower ALB compared to survivors. Our data agree with several works that describe hypoalbuminaemia as a negative prognostic factor during PLE and IRE [7,9,10,24]. In our opinion, as reported in human medicine in patients with IBD, it is possible that protein and lipidic malabsorption at the time of diagnosis may reduce a dog’s ability to respond to therapies [11,25,26].

A novel and interesting finding in our study is the reduction of BCS in non-responders and in non-survivors. These data may be linked to the systemic involvement of intestinal inflammation, and they may be connected to a functional intestinal insufficiency, as reported in humans. In human medicine, weight loss and malnutrition worsen the prognosis and represent an important co-morbidity associated with complications in IBD patients [25,26,27,28]. In veterinary medicine, only a few studies have evaluated BCS in dogs with chronic enteropathies, specifically in PLEs [11,29], and the BCS was not associated with mortality.

Our study also highlights the importance of histopathological morphological lesions, and the presence of LD seems especially to be linked to mortality and relapse in 18 months follow-up. Allenspach et al. [30], in a retrospective study, showed an association between clinical scores (CIBDAI/CCECAI) at the time of diagnosis and duodenal histopathological lesions (CD, MF, LD, villous atrophy, neutrophilic infiltrate and the presence of lymphocytes and plasma cells in the lamina propria). However, the clinical progress and follow-up of these patients were not evaluated. Furthermore, in Wennogle’s study on dogs with chronic enteropathies, hypoalbuminaemia was associated with the presence of histopathological lesions, such as villous atrophy, CD, LD, IEL and increased neutrophils in the lamina propria [31]. In addition, Moser found an association between the increase in IEL and high CIBDAI and hypoalbuminaemia [32]; however, in our cases, these histopathological alterations did not seem to be associated with a PLE. Although the severity in inflammatory infiltrate does not indicate the severity of the intestinal disease, the structural/histologic alterations in the intestinal villi seem to play a predominant role [30,31]. Wennogle et al. reported that dogs with PLE were significantly more likely to display villous stunting, epithelial injury, CD and LD, and to have IEL and lamina propria neutrophils than dogs without hypoalbuminaemia (non-PLE). To date, no works have evaluated histopathological lesions and the long-term follow-up of dogs with IRE.

Our results would seem to indicate that integrating the WSAVA histopathological score with a characterization of intestinal structural morphological lesions, it possible to provide the clinical behaviour of dogs affected by IRE in the relapse and the long-term follow-up. Based on our results, the presence of LD may be associated with severe damage to the intestinal wall, which can be the cause of a lack of response and relapse during follow-up. For this reason, it may be important to evaluate LD together with hypoalbuminaemia.

In our population, endoscopic and histological evaluations were performed in all dogs, although we considered only the histopathological evaluation of the duodenum. This was not true for the ileum, which might be because of the small number of dogs in which ileal biopsies were performed. Additionally, standardized scoring of both endoscopy and histology findings specifically for the ileum is not included in the WSAVA guidelines [18,33], even though the scoring system used for duodenal biopsies has been previously validated for ileal lesions [30].

This study has some limitations. To make a diagnosis of IRE, diet-responsive enteropathies were excluded using a diet trial. Based on the recent study of Nagata et al. on dogs with hypoalbuminaemia, a diet trial with an ultra-low-fat food may be recommended [16]. In our study a hydrolysed diet with low fat was performed in many dogs with hypoalbuminaemia (HA Purina^®^, Nestlé Purina S.p.a. Assago, (MI) Italy). However, this prospective study began in 2016, and this ultra-low-fat diet trial was not performed in all dogs, and for this reason, we cannot exclude that our population included food-responsive PLE.

Moreover, since guidelines have been established on the choice of cut-offs to evaluate the response to therapy and relapse, we followed other authors and chose them both [10,14]. Another limitation is the non-uniformity of the immunomodulatory treatment between various cases, which might have influenced the response to therapy and relapse. However, although there is no consensus on IRE treatment in dogs, in our study, most dogs had received prednisolone as a first line, with or without cyclosporine, and as a second line, chlorambucil with or without cyclosporine. In addition, since IRE is chronic disease without a standard therapy, clinicians have to monitor the case and modulate the therapy according to the clinical response along with any adverse drug reactions that may occur during follow-up. Lastly, since information regarding follow-up was lost in 10% of our dogs, death may possibly have occurred, since they were not monitored. Another limitation is linked to the non-survivors. This group had to also include dogs that were euthanized, even if no dogs were euthanized due to financial concerns or due to causes unrelated to IRE/NRE, but only for the deterioration of clinical condition despite treatment. The lack of a supervisor for endoscopic and histopathological reports may represent another limitation. Even if there a was not a double-blind review of the histopathological samples, all the experienced operators followed the WSAVA guidelines for histology interpretation. In addition, in the present study was only evaluated the presence/absence of single histopathological lesions. Considering the number of cases included and different pathologists involved, we decided to avoid the use of a potentially subjective score on histopathological lesions severity.

Moreover, the number and characterization of dogs enrolled at each centre could be another potential covariate in the analysis. In addition, an immunohistochemistry (IHC) or PCR for antigen receptor rearrangements (PARR) was not performed in our population. Recently, two studies described low-grade lymphoma in dogs that underwent endoscopic evaluation for gastrointestinal signs [34,35]. In our cases, it was not possible to rule out that in the NRE group there were included also low-grade lymphoma. In our study, the presence of ARE was ruled out using an antibiotic trial. Recently, the usefulness of antibiotic trials after diet trials was called into question by Cerquetella [2], who proposed novel inclusion criteria in the diagnosis of IRE, and thus we cannot exclude that the antibiotic trial may have influenced our results.

## 5. Conclusions

This study shows that IRE was an intestinal disease with a good clinical course in most of the patients. The non-response or relapse rate varied between 9 and 11%, respectively. The evaluation of BCS, ALB and the presence of histopathological LD at the time of diagnosis seems to predict the response to therapy, the mortality and the long-term disease control.

## Figures and Tables

**Table 1:** Summary of abbreviations, time of evaluation, variables type and class of the variables included in the study.

Variable	Abbreviation	Time	Variable Type	Variable Classes	Outcome or Potential Predictor
Age		T0	Continuous		Potential predictor
Sex		T0	Categorical nominal	Female Male	Potential predictor
Sexual status		T0	Categorical nominal	Entire De-sexed	Potential predictor
Body condition score	BCS	T0	Categorical ordinal	1–3; 4–6.	Potential predictor
Total protein	TP	T0	Continuous	N/A	Potential predictor
Albumin	ALB	T0	Continuous	N/A	Potential predictor
Cholesterol	COL	T0	Continuous	N/A	Potential predictor
C-reactive protein	CRP	T0	Continuous	N/A	Potential predictor
Tissue appearance at endoscopy		T0	Categorical ordinal	Normal, 0; Mild, 1; Moderate, 2; Severe, 3	Potential predictor
Tissue histopathology		T0	Categorical ordinal	Normal, 0; Mild, 1; Moderate, 2; Severe, 3	Potential predictor
Crypt dilatation	CD	T0	Categorical nominal	Yes/no	Potential predictor
Intraepithelial lymphocytes	IEL	T0	Categorical nominal	Yes/no	Potential predictor
Mucosal fibrosis	MF	T0	Categorical nominal	Yes/no	Potential predictor
Lacteal dilatation	LD	T0	Categorical nominal	Yes/no	Potential predictor
Sum of histopathological lesions	SUM	T0	Categorical ordinal	Sum of scores for presence/absence of crypt dilatation (CD), intraepithelial lymphocytes (IEL), mucosal fibrosis (MF) and lacteal dilatation (LD)	Potential predictor
Canine chronic enteropathy clinical activity index at T0	CCECAIT0	T0	Categorical ordinal	0–9; >=10	Potential predictor
Response to treatment		T1 vs. T0	Categorical ordinal	Complete response: reduction in CCECAI >75% Partial response: reduction in CCECAI of 25–75% No response: reduction in CCECAI <=25%	Outcome
Relapse		T3 > T18	Categorical nominal	Yes/no, with yes defined as either an increase of >=2 in the CCECAI or death or euthanasia between any 2 time points from T3 to T18	Outcome
Mortality		T1–T18	Categorical nominal	Yes/no with yes defined as either death or euthanasia	Outcome
Long-term response		T1–T18	Categorical nominal	Positive or negative where positive is defined as dogs responding at T0 and with no subsequent relapse, death or euthanasia	Outcome

**Table 2:** Descriptive statistics of included dogs with population sample characterization and blood parameters.

Variable		
Age	4 (7–15 Years)	
Sex and Sexual Status	100 Males 65 Females	100 Intact Males
		33 Spayed Females, 32 Intact Females
Breed	Mixed-breed	36 dogs (22%)
	German Shepherd	26 dogs (16%)
	Boxer	8 dogs (5%)
	English Setter	7 dogs (4%)
	Yorkshire Terrier	6 dogs (3%)
	Jack Russell Terrier	6 dogs (3%)
	Dachshund	5 dogs (3%)
	Maltese	5 dogs (3%)
	Border Collie	4 dogs (2%)
	Pinscher	4 dogs (2%)
	Rottweiler	4 dogs (2%)
	Golden Retriever	3 dogs (2%)
	Siberian Husky	3 dogs (2%)
	Bernese Mountain Dog, Italian Bracco, Cavalier King Charles Spaniel, Chihuahua, Dobermann, Irish Setter, Labrador Retriever, Poodle, Pug, Springer Spaniel, Weimaraner and West Highland White Terrier	2 dogs each breed
	Akita Inu, Great Dane, Australian Shepherd, Basenji, Beagle, Belgian Shepherd, Bichon Frise, Bolognese, Central Asian Shepherd, Cocker Spaniel, Cane Corso, Dogue de Bordeaux, English Bulldog, Lagotto Romagnolo, Parson Russell Terrier, Russian Toy, St. Bernard, Shih Tzu, Pitbull, Levriero Italiano, Spanish Galgo, Swiss Shepherd Dog, Vizsla and Italian Volpino	1 dog each breed
Body condition score	4 (range 1–6)	BCS 1/9 → 1 dog (0.6%)
		BCS 2/9 → 9 dogs (5.5%)
		BCS 3/9 → 54 dogs (32.7%)
		BCS 4/9 → 65 dogs (39.4%)
		BCS 5/9 → 34 dogs (20.6%)
		BCS 6/9 → 2 dogs (1.2%)
Total protein	5.5 ± 1.3 g/dL	Hypoproteinaemia → 92 dogs (56%)
Albumin	2.6 g/dL (range 0.7–4.4 g/dL)	Hypoalbuminaemia (PLE) → 84 dogs (51%)
Cholesterol	152 mg/dL (range 54–428 mg/dL)	Hypocholesterolaemia → 42 dogs (25%)
C-reactive protein	0.3 mg/dL (range 0–2.8 mg/dL)	↑ CRP (>0.3 mg/dL) → 81 dogs (49%)
Endoscopic score	Endoscopic score 0–1 → 0 dogs	
	Endoscopic score 2 → 66 dogs (40%)	
	Endoscopic score 3 → 99 dogs (60%)	
Histopathological score	Histopathological score 1 → 11 dogs (7%)	
	Histopathological score 2 → 108 dogs (65%)	
	Histopathological score 3 → 46 dogs (28%)	
Histopathological lesions	Crypt distension	115 dogs (70%)
	Intraepithelial lymphocytes	64 dogs (39%)
	Mucosal fibrosis	45 dogs (27%)
	Lacteal dilatation	62 dogs (39%)

**Table 3:** Correlations between clinical and haematological variables at admission in 165 dogs with immunosuppressant-responsive and non-responsive enteropathies.

Parameter	BCS	TP	ALB	COL	CRP	SUM	CCECAI
BCS	1	*p* = 0.0001 ρ = 0.317	*p* = 0.0001 ρ = 0.362	*p* = 0.005 ρ = 0.218	NS	*p* = 0.0001 ρ = −0.240	*p* = 0.012 ρ = −0.199
TP		1	*p* = 0.0001 ρ = 0.867	*p* = 0.0001 ρ = 0.649	NS	*p* = 0.0001 ρ = −0.258	NS
ALB			1	*p* = 0.0001 ρ = 0.582	NS	*p* = 0.002 ρ = −0.240	NS
COL				1	*p* = 0.014 ρ = 0.193	*p* = 0.047 ρ = −0.156	NS
CRP					1	NS	NS
SUM						1	NS
CCECAI							1

ALB, albumin; BCS, body condition score; CCECAI, Canine Chronic Enteropathy Clinical Activity Index; COL, cholesterol; CRP, C-reactive protein; ns, not significant; SUM, sum of histopathological lesions; TP, total protein; NS, not significant (*p* > 0.05).

## Data Availability

The data presented in this study are available upon request from the corresponding author. The data are not publicly available due to other project involvement.

## Supplementary Materials

The following are available online at https://www.mdpi.com/article/10.3390/ani11092637/s1, Table S1: Scheme on how to calculate Canine Chronic Enteropathy Activity Index (CCECAI) [7].
