# Supplementary material for: Immunosuppressant-Responsive Enteropathy and Non-Responsive Enteropathy in Dogs: Prognostic Factors, Short- and Long-Term Follow Up
Source: Animals (Basel). 2021 Sep 8;11(9):2637. doi: 10.3390/ani11092637 (PMC8472317; doi:10.3390/ani11092637)
Supplement: Supplementary file 1 [file animals-11-02637-s001.zip › animals-1326896-supplementary.pdf]

Table S1. Scheme on how to calculate Canine Chronic Enteropathy activity index (CCECAI) [7].

Attitude/activity	0 normal<br>1 slightly decreased<br>2 moderately decreased<br>3 severely decreased
Appetite	0 normal<br>1 slightly decreased<br>2 moderately decreased<br>3 severely decreased
Vomiting	0 normal<br>1 mild (< 1 event/week)<br>2 moderate (<2–3 event/week)<br>3 severe (> 3 events/week)
Fecal consistency	0 normal<br>1 slightly soft feces<br>2 very soft feces<br>3 watery diarrhea
Fecal frequency	0 normal<br>1 slightly increased (< 2 events/day) or<br>fecal blood, mucus, or both<br>2 moderately increased (< 4–5 events/day)<br>3 severely increased (> 5 events/day)
Weight loss	0 none<br>1 mild (<5%)<br>2 moderate (5–10%)<br>3 severe (>10%)
Albumin levels	0 albumin >20g/L<br>1 albumin 15–19.9 g/L<br>2 albumin 12–14.9 g/L<br>3 albumin <12 g/L
Ascites and peripheral edema	0 none<br>1 mild ascites or peripheral edema<br>2 moderate amounts of ascites/ peripheral edema<br>3 severe ascites/pleural effusion and peripheral edema
Pruritus	0 no pruritus<br>1 occasional episodes of itching<br>2 regular episodes of itching,<br>but stops when dog is asleep<br>3 pruritis regularly wakes dog
